# Intrathecal injections of non-peptide oxytocin receptor agonists, WAY 267,464 and TC OT 39, induce significant anti-hyperalgesia in both male and female rats with inflammatory pain

**DOI:** 10.22038/ijbms.2025.86093.18615

**Published:** 2025

**Authors:** Lok-Hi Chow, Yuan-Hao Chen, Ying-Jie Chen, Pin-Chen Lin, Eagle Yi-Kung Huang

**Affiliations:** 1 Department of Anesthesiology, Taipei Veterans General Hospital, Taipei, Taiwan; 2 Department of Anesthesiology, School of Medicine and Institute of Clinical Nursing, School of Nursing, National Yung Ming Chiao Tung; 3 University, Taipei, Taiwan; 4 Department of Anesthesiology, National Defense Medical Center, Taipei, Taiwan; 5 Department of Pharmacology, National Defense Medical Center, Taipei, Taiwan; 6 Department of Neurological Surgery, Tri-Service General Hospital, National Defense Medical Center, Taipei, Taiwan

**Keywords:** Hyperalgesia, Insulin-regulated- aminopeptidase, Oxytocin, Oxytocin receptor, Sex difference

## Abstract

**Objective(s):**

Hyperalgesia is a clinical condition related to chronic pain in which patients experience increased nociceptive sensitivity. Intrathecal oxytocin has been shown to induce significant anti-hyperalgesia in both rodents and humans. However, in our previous studies, we demonstrated a clear sex difference in oxytocin’s effects at the spinal level in rodents. We suggested that this sex difference could be partially due to the higher expression of the oxytocin-degrading enzyme, insulin-regulated aminopeptidase (IRAP), in the spinal cord of females. Thus, we aimed to evaluate non-peptide oxytocin receptor (OTR) agonists, which we speculated could effectively reduce inflammatory hyperalgesia in both sexes due to their resistance to IRAP degradation.

**Materials and Methods:**

Plantar tests were conducted on adult S.D. rats of both sexes to examine intraplantar carrageenan-induced hyperalgesia. This was followed by an intrathecal (i.t.) injection of non-peptide OTR agonists, WAY 267,464 and TC OT 39, to assess their effects on hyperalgesia. Atosiban, an OTR antagonist, was also co-administered with these compounds to confirm their involvement in OTR activation.

**Results:**

WAY 267,464 and TC OT 39 were both effective in ameliorating anti-hyperalgesia in male and female rats, suggesting no sex difference. Additionally, atosiban attenuated the anti-hyperalgesia effects of WAY 267,464 and TC OT 39, confirming these compounds’ involvement in OTR activation.

**Conclusion:**

These compounds induced significant anti-hyperalgesia without any sex differences, suggesting that the inhibition of IRAP degradation eliminated the variation in oxytocin’s effects based on sex. Therefore, we propose these compounds as promising candidates for treating inflammatory hyperalgesia in clinical applications.

## Introduction

Oxytocin, released from the posterior pituitary, was found to have many physiological functions and plays roles in social bonding, reproduction, and childbirth (1). Notably, it was found to be a novel endogenous substance that regulates nociception (2). Condes-Lara *et al*. have identified a novel oxytocin neural pathway originating from the paraventricular nucleus and projecting to the dorsal horn of the spinal cord (3). This neural pathway has been proven to regulate nociception. It is also indicated to be released by hypothalamospinal fibers terminating in lamna I–II of the spinal dorsal horn and activating glutamatergic neurons (4). The population of oxytocin-sensitive glutamatergic neurons recruits the whole population of GABAergic neurons, resulting in the elevated GABAergic inhibitory tone to cause antinociception at the spinal level. Yu *et al*. reported an involvement of the endogenous opioid system in oxytocin-induced antinociception in the spinal cord of rats with inflammation (5). Our previous study further confirmed the role of oxytocin in inflammatory hyperalgesia. We showed that intrathecal (i.t.) oxytocin administration attenuated inflammatory hyperalgesia in adult male rats (6, 7). 

Many chronic pain conditions can induce hyperalgesia and allodynia, e.g., complex regional pain syndrome (CRPS)(8). Hyperalgesia, specifically, refers to an increased pain response to a stimulus that usually provokes pain (9). Literature suggests that its underlying mechanisms involve increased descending facilitation in the spinal cord, which is involved in chronic pain conditions (10). Because hyperalgesia is a medical condition that causes an increased sensitivity to pain, patients are always suffering and eager to be treated for relief. However, several medications may not have satisfactory therapeutic effects for hyperalgesia. Therefore, oxytocin and related compounds could be developed into novel drugs for pain treatment. Its effectiveness has also been verified in some clinical studies. Yang and Condés-Lara *et al*. proved the positive effect of oxytocin in reducing low back pain and cancer pain (11, 12). These reports revealed a great potential for the application of oxytocin-related drugs in pain management.

Nevertheless, our series of studies revealed a sex difference in oxytocin-induced anti-hyperalgesia and anti-allodynia in rodents (6, 7). Intrathecal injections of oxytocin proved effective in male animals but not in females at equivalent doses. Moreover, we found that the expression of insulin-regulated aminopeptidase (IRAP), the primary enzyme responsible for oxytocin degradation, was higher in the spinal cord of female rats (6). We suggested that this can lead to a faster degradation of oxytocin, which results in the absence of oxytocin’s effect in female rats. In this context, we attempted to look for some non-peptide oxytocin receptor (OTR) agonists that can escape IRAP degradation.

In the present study, two compounds, WAY 267,464 and TC OT 39, were used. WAY 267,464 was a potent and selective non-peptide agonist for OTR (13). Hicks *et al*. have performed binding displacement of WAY 267,464 on OTR and vasopressin receptor 1A (V_1a_R) expressed on HEK293 cells (14). Its binding affinity to OTR was about 1,000 times lower than that of oxytocin, as revealed by the Ki value. However, its selectivity was higher and had a lower affinity for V_1a_R. Several studies have utilized WAY 267,464 as an OTR agonist and tested for the effects induced by OTR activation, including fear acquisition and extinction, social behavior, body temperature, novelty-induced hypophagia, circadian rhythms, social recognition memory, and social anxiety following ethanol exposure (15-21). TC OT 39 acts as a partial agonist of the oxytocin and vasopressin V2 receptors (V_2_Rs) (Ki = 147 nM and >1,000 nM, respectively) and antagonist of the vasopressin 1A receptor (Ki = 330 nM) (22, 23). The binding affinities and activities of oxytocin, WAY 267,464 and TC OT 39, on OTR, V_1a_R, and V_2_R are listed in Table 1.

To assess their effectiveness, intrathecal injection of these agonists was used to determine their effects on inflammatory hyperalgesia in rats using the intraplantar carrageenan model. To examine the role of IRAP expression in the spinal cord in sex differences, non-peptide agonists of the oxytocin receptor (OTR) were also tested for comparison in both sexes of rats. Moreover, the co-administration of atosiban (an OTR antagonist) is used to confirm that the anti-hyperalgesia produced by non-peptide OTR agonists acts through OTR.

## Materials and Methods

### Animals

Adult male and female Sprague–Dawley rats (8-week-old male/virgin female rats) were purchased from BioLASCO Taiwan Co., Ltd. The body weights of male rats were between 300 g and 400 g. Female rats were from 300 g to 400 g. All animals were kept in the animal house of the National Defense Medical Center, Taipei, Taiwan. The animal rooms were maintained at 23±2 ^°^C in a 12-hr light/dark cycle, and food and water were provided *ad libitum *throughout the experiments. Animals were transferred to the testing room to adapt to the environment for two hours per day for three days before the commencement of behavioral experiments. The experimental protocols were approved by the Animal Care and Use Committee of the National Defense Medical Center, Taipei, Taiwan (reference no. IACUC-19-156). 

### Animal groups

The male rats were randomly divided into 8 groups and treated with different agents, whereas the female rats were randomly divided into 7 groups and treated with different agents (Table 2).

### Drugs

WAY-267,464 dihydrochloride (cat. No. 3933) and TC OT 39 (cat. No. 4625) were purchased from Tocris, UK. Both compounds are non-peptide OTR agonists and V_1a_R antagonists. WAY-267,464 was dissolved in normal saline (0.9% NaCl) and diluted to the desired concentration for intrathecal injection. Due to its lower solubility, TC OT 39 was first dissolved in 100 mM HCl and then further diluted with saline to the required concentration. After dilution, the final concentration of HCl for injections was lower than 125 µM. The doses of WAY 267,464 were 0.125, 1.25, 12.5, and 50 nmol per rat. Only one dose of TC OT 39 at 12.5 nmol was used. Based on our previous studies, these dosages were chosen, where oxytocin was administered intrathecally at a dose of 0.125 nmol (24). Pentobarbital was purchased from SCI Pharmtech Inc. and administered at a dose of 60 mg/kg via intraperitoneal (IP) injection. Atosiban was obtained from PROSPEC (Cat. No. hor-239-a) and used at 12.5 nmol, a dose equivalent to that of the oxytocin receptor agonists.

### Implantation of intrathecal catheters

As described in our previous report, the same intrathecal (i.t.) catheter implantation method in rats was used (25, 26). After the rats were anesthetized with pentobarbital (60 mg/kg, IP), an intrathecal catheter was implanted at the lumbar level (L5-L6) for drug administration. To ensure that the catheters reached the same position in both sexes of rats, the one used for female rats was 2 mm shorter than that used for male rats. The animals were allowed to recover from surgery for 5 days. No animal was used in more than one experiment. One day before the experiment, animals bearing the i.t. catheter were injected with 20 µl of 2% lidocaine using a microsyringe (Hamilton, 25 µl) to induce a temporary motor blockade of the lower limbs to determine whether the catheter was positioned correctly.

### Plantar test in carrageenan-induced inflammation

To induce acute inflammation, 0.1 ml of carrageenan type IV (Sigma, USA) solution (1.5%, w/v in saline) was injected into the subcutaneous space of the right hind paw**. **Following carrageenan injection, the test compounds were injected i.t. into the rats through the catheter. A Ugo Basile (Italy) 7371 plantar tester was used to measure the withdrawal latency of the carrageenan-injected paw. The IR intensity was set at 60 (150 mW/cm^2^), and the cutoff time was 20 s. Basal latency was determined before the intraplantar injection of carrageenan (-1 h), and the latencies were measured after the injection at 0, 1, 2, 3, 4, 4.5, 5, 5.5, 6, 7, 24, and 31 hr, which was based on our previous studies (25, 27)

### Statistical analysis

All data were presented as means±SEM. Two-way ANOVA followed by Bonferroni *post hoc* test was used to examine the significant differences between groups. *P*-values<0.05 were considered statistically significant. To evaluate the anti-hyperalgesic effects of WAY 267,464 and TC OT 39 on nociception, the area under the curve (AUC) (-1 to 7 h) of each curve was calculated as nociceptive scores using the linear trapezoidal method. One-way ANOVA followed by Tukey’s multiple comparison test was used to compare all groups.

## Results

### WAY 267,464 (i.t.) dose-dependently induced a significant anti-hyperalgesia in male rats

**Figure 1:**
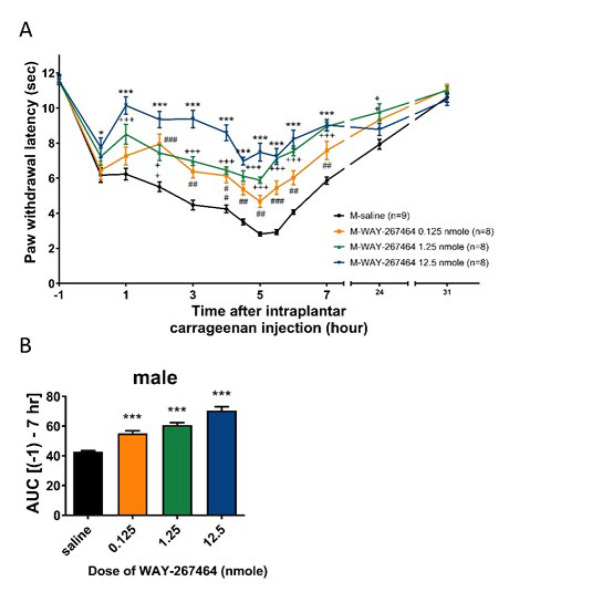
Effects of intrathecally injected WAY 267,464 on thermal hyperalgesia induced by intraplantar carrageenan injections in male rats

The basal paw withdrawal latency in male rats was 11.2±0.2 sec. In the saline group, the latencies decreased to 6.2±0.4 s at 0 hr (measured promptly following carrageenan injection), possibly due to acute pain during injection. At 1 to 5 hr, the latencies gradually decreased to a minimum of 2.8±0.1 sec. Over time, it increased and returned to the basal value. At 31 hr, the values returned to 10.6±0.3 sec, which is consistent with the results of our previous report (6). At a dose of 0.125 nmol, WAY 267,464 significantly increased paw withdrawal latency at 2 hr (7.9±0.6 sec). At 5 hr, the latencies reached the minimum values (4.7±0.3 sec), significantly higher than those obtained from the saline group. The latencies (11.1±0.3 sec) returned close to the basal value. In the WAY 267,464 groups at doses of 1.25 nmol and 12.5 nmol, the same pattern of hyperalgesia inhibition was observed, but with a higher extent of inhibition compared to a dose of 0.125 nmol. The minimum latency values decreased to 5.9±0.2 s (at 5 hours) and 7.0±0.2 s (at 4.5 hours), respectively. When compared with the values of the saline group, the minimum latency values were both higher. The curves are shown in Figure 1A. To quantify the anti-hyperalgesic effects of WAY 267,464, each curve’s AUC (-1 to 7 h) was calculated for comparison (Figure 1B). Data showed that WAY 267,464 induced significant anti-hyperalgesic effects in all tested doses. The extent of anti-hyperalgesia was likely to be dose-dependent.

### WAY 267,464 (i.t.) also induced significant anti-hyperalgesia in female rats, compared with that in male rats

**Figure 2:**
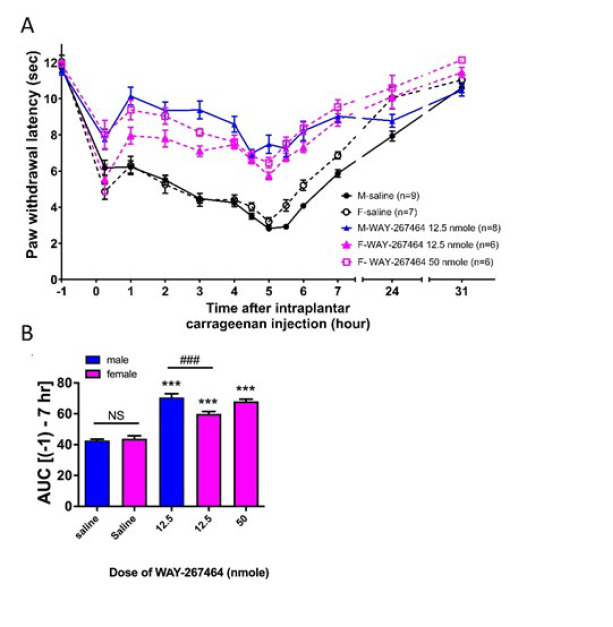
Effects of intrathecally injected WAY 267,464 on thermal hyperalgesia induced by intraplantar carrageenan injections in male and female rats

Figure 2A shows the curves of the effects of WAY 267,464 on hyperalgesia. The basal paw withdrawal latencies in female rats were approximately 12 sec, similar to those observed in male rats. WAY 267,464 (i.t.) was administered to female rats at the highest dose (12.5 nmol) tested in male rats. At this dose, WAY 267,464 also induced a significant anti-hyperalgesic effect in female rats. Five hours after carrageenan injection, the latencies decreased to 3.2±0.2 sec in the female saline group, whereas the latencies were 5.7±0.2 sec in the female WAY 267,464 12.5 nmol group. However, this anti-hyperalgesia was significantly lower than that in male rats. When a higher dose of 50 nmol was administered to female rats, a slightly higher anti-hyperalgesia effect was observed (minimal latency at 5 h: 6.5±0.3 sec) than that of the 12.5 nmol group. However, this anti-hyperalgesia was still not higher than that in WAY 267,464 12.5 nmol-induced anti-hyperalgesia in male rats. The AUC (-1 to 7 h) was calculated to quantify the extent of hyperalgesia, as shown in Figure 2B. To compare the extent of hyperalgesia in male and female rats in the saline group, no sex difference was observed when the animals received the same dose of carrageenan injection. The AUC results showed that in the WAY 267,464 12.5 nmol group, the AUC in female rats was significantly lower than in male rats at the same dose. When the dose was increased to 50 nmol in female rats, there was no significant difference compared to male rats at the 12.5 nmol dose. However, the AUCs of WAY 267,464 12.5 nmol and 50 nmol groups of female rats were both higher than those of the saline group, suggesting that WAY 267,464 remained effective in inducing anti-hyperalgesia in female rats, with lower efficacy compared to the male rats.

### TC OT 39 (i.t.) induced a significant anti-hyperalgesia in both male and female rats

**Figure 3:**
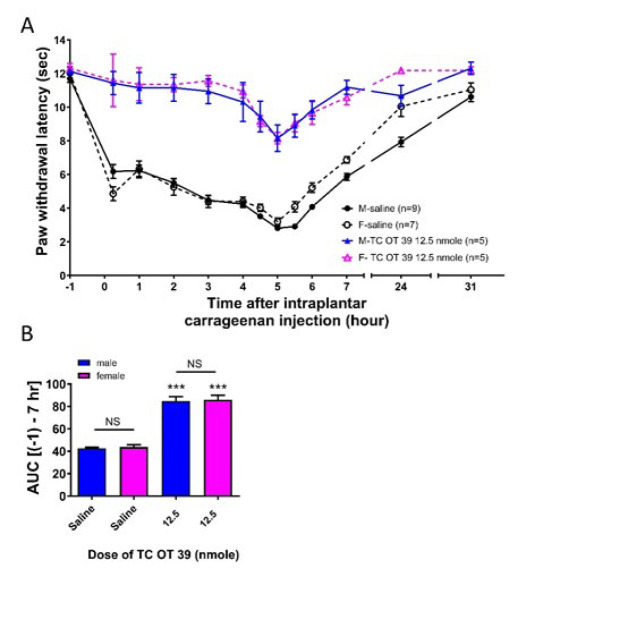
Effects of intrathecally injected TC OT 39 on thermal hyperalgesia induced by intraplantar carrageenan injections in male and female rats

As shown in Figure 3A, TC OT 39 (i.t.) was tested in male and female rats at a dose of 12.5 nmol. TC OT 39 induced significant anti-hyperalgesia in both male and female rats. The extent of anti-hyperalgesia induced by TC OT 39 was not different between male and female rats. At 5 hr, TC OT 39 increased the paw withdrawal latencies to 8.2±0.8 sec and 8.2±0.3 sec in male and female rats, respectively. The values of the corresponding saline groups were 2.8±0.1 sec and 3.2±0.2 sec, respectively. AUC data (Figure 3B) showed that TC OT 39 induced a significant anti-hyperalgesic effect in male and female rats. These effects were not associated with sex differences. 

### Atosiban decreased the anti-hyperalgesia effects produced by WAY 267,464 and TC OT 39 in rats

**Figure 4:**
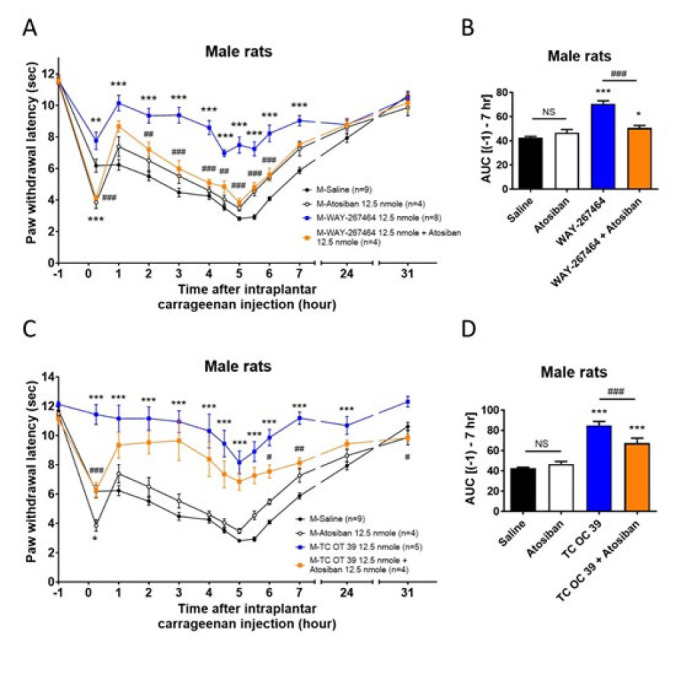
Effects of the co-administration of atosiban with WAY 267,464 or TC OT 39 on thermal hyperalgesia induced by intraplantar carrageenan injections in male rats

**Figure 5:**
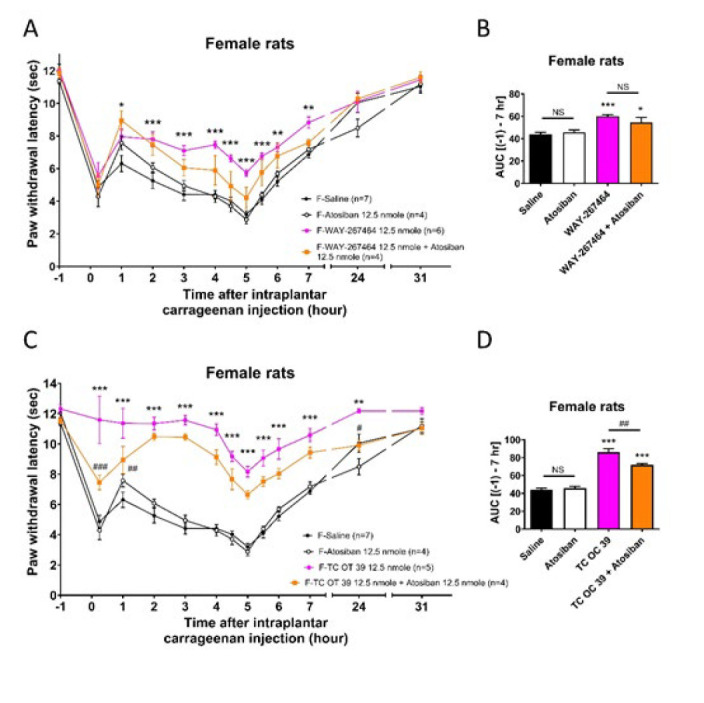
Effects of co-administration of atosiban with WAY 267,464 or TC OT 39 on thermal hyperalgesia induced by intraplantar carrageenan injections in female rats

Figures 4 and 5 show that co-administration of atosiban (i.t.) with these two drugs was tested in male and female rats at 12.5 nmol. In male rats, the anti-hyperalgesia curves (Figure 4A, Figure 4C) induced by WAY 267,464 and TC OT 39 were significantly reduced in the groups that received co-administration of atosiban. The extent of AUC (Figure 4B, Figure 4D) further showed that co-administration of atosiban significantly blocked the anti-hyperalgesic effects of both WAY 267,464 and TC OT 39 in male rats. In female rats (Figure 5), the curves (Figure 5A) showed an attenuation trend in the anti-hyperalgesia effect of WAY 267,464 when co-administered with atosiban. However, the AUC results (Figure 5B) indicated that the extent of anti-hyperalgesia induced by WAY 267,464 was not significantly affected by atosiban, suggesting that other significant mechanisms might be involved in the WAY 267,464-induced anti-hyperalgesia. Conversely, the anti-hyperalgesia effect caused by TC OT 19 was significantly decreased after co-administration with atosiban (Figures 5C and 5D). Nevertheless, its effect was only partial compared to that of the saline group, indicating that other mechanisms might also contribute. These results suggested that WAY 267,464 and TC OT 39 act primarily through the activation of OTR to induce their anti-hyperalgesia effects in male rats. However, other effects on the unidentified targets may also be involved in female rats, especially for WAY 267,464.

## Discussion

Our previous study demonstrated that intrathecal oxytocin injection significantly induced anti-hyperalgesia in a rat inflammation model caused by intraplantar carrageenan injection (6). We found that the effect of oxytocin was notably significant in male rats, with the paw withdrawal latency extending beyond the baseline value at a dose of 0.125 nmol in the plantar test. However, at the same dose of 0.125 nmol, oxytocin did not induce anti-hyperalgesia in female rats. When the dose was increased to 1.25 nmol, oxytocin significantly induced anti-hyperalgesia in female rats. This oxytocin-induced anti-hyperalgesia was similar to that observed in male rats. These results suggest that oxytocin is 10 times less potent in inducing anti-hyperalgesia in female rats than in male rats. Additionally, we have confirmed that in both sexes of animals, the effect of oxytocin was significantly blocked by the co-administration of the selective OTR antagonist, atosiban. This suggests that the activation of OTR mainly caused the anti-hyperalgesia caused by oxytocin, but not vasopressin receptors. This study attempted to identify the mechanisms underlying the sex difference in oxytocin-induced anti-hyperalgesia at the spinal level. It was found that IRAP (an oxytocin-degrading enzyme) plays an important role. IRAP expression was higher in the spinal cords of female rats when compared with that of male rats (6). This implies a more substantial and faster degradation of oxytocin in female spinal cords, which leads to the absence of oxytocin’s effects. Our recent report further proved that estrogen may also play a role in this sex difference (24).

To check whether IRAP expression has a functional impact on this sex difference, two non-peptide OTR agonists were applied, WAY 267,464 and TC OT 39, in the same animal model of inflammatory hyperalgesia. Because these two compounds should be resistant to IRAP degradation, we speculate that they should effectively induce anti-hyperalgesia with no differences between sexes. Our results showed that both compounds required higher dosages to achieve similar anti-hyperalgesia effects as oxytocin. WAY 267,464 became effective at a dose of 0.125 nmol in male rats, but the efficacy of anti-hyperalgesia was much lower than that of oxytocin. When the dose reached 12.5 nmol, the efficacy of WAY 267,464 was still lower. This is in agreement with the OTR binding affinity of WAY 267,464. As shown in Table 1, the K_i_ value of WAY 267,464 was 1,000 times higher than that of oxytocin, suggesting a much lower binding affinity to OTR. Although the testing dose of WAY 267,464 could not be increased to 125 nmol (1,000 times) owing to its solubility, our data revealed the effectiveness of WAY 267,464 in inducing a satisfactory anti-hyperalgesia at a high dose in male rats. As expected, WAY 267,464 also induced significant anti-hyperalgesia at 12.5 nmol in female rats. Although the extent of the effect was lower than that observed in male rats at the same dose, increasing the dose to 50 nmol did not result in a significant difference compared to the 12.5 nmol dose in male rats. This result indicates that WAY 267,464 is effective in both sexes, albeit with relatively lower efficacy in female rats. This implies that the use of WAY 267,464 should have fewer sex differences to limit its application.

TC OT 39 also showed a clear anti-hyperalgesia at the 12.5 nmol dose in both sexes of rats. The extent was higher than that of WAY 267,464 at the same dose. The OTR binding affinity of TC OT 39 was approximately seven times higher and 147 times lower than those of WAY 267,464 and oxytocin, respectively. This result was consistent with the results of the binding assays. Moreover, the sex difference was also absent in the results of TC OT 39. Our data demonstrate that TC OT 39 might hold potential therapeutic value for treating inflammatory hyperalgesia. As mentioned, oxytocin exerts anti-hyperalgesia via OTR activation. Nevertheless, oxytocin is known to bind to V_1a_R and act as an agonist (28). In contrast, WAY 267,464 and TC OT 39 were both proven to be agonists of OTR and antagonists of V_1a_R (20, 23). Although the blockade of the effects of endogenous vasopressin on V_1a_R may have a minor influence, the amount of vasopressin in the spinal cord is much less than oxytocin. Thus, the anti-hyperalgesia of these two compounds should come mainly from OTR activation. However, further investigation is needed to validate. The V_2_R binding affinity of these two non-peptide OTR agonists was very low and could be neglected. Both WAY 267,464 and TC OT 39 can induce a significant anti-hyperalgesia effect intrathecally. However, TC OT 39 could be better than WAY 267,464 for a new drug development since WAY 267,464 still had a slightly lower potency and efficacy in female rats. The possible mechanisms underlying the difference between WAY 267,464 and TC OT 39 require further investigation, apart from the binding affinity and selectivity to the receptors. 

Most importantly, we used atosiban, an OTR antagonist, to demonstrate that the anti-hyperalgesia effects produced by these drugs are mainly through the activation of OTR. Notably, our findings revealed that atosiban effectively blocked the anti-hyperalgesia effects induced by TC OT 39 in both male and female rats. However, while atosiban inhibited the anti-hyperalgesia effects of WAY 267,464 in male rats, it failed to block these effects in female rats significantly. This implies that the anti-hyperalgesia action of TC OT 39 predominantly involves OTR activation in both sexes of rats. In contrast, other mechanisms might play a significant role in the pain relief provided by WAY 267,464 in female rats.

It is noteworthy that current evidence suggests atosiban functions as an antagonist for Gq-coupled OTR and a biased agonist for G_i_-coupled OTR. This dual function of atosiban sheds light on its inability to block the anti-hyperalgesia effects of non-peptide OTR agonists consistently. The efficacy of the blockade of atosiban may depend on the primary action of the agonist, targeting either Gq- or Gi-coupled OTR, or potentially both. Another possible explanation is that the expression of Gq- or Gi-coupled OTR receptors might differ between male and female rats; however, further experiments are needed to validate this. A previous study also revealed that the analgesia effect of oxytocin resulted from V1aR receptor rather than OTR using knockout mice, indicating the importance of the V1aR receptor in the analgesia effect (29). However, using both drugs as V1aR receptor antagonists effectively excludes the involvement of the V1aR receptor. Our results confirmed that their analgesia effects come from OTR by using the OTR antagonist, atosiban. Nevertheless, in addition to using the oxytocin receptor antagonist, it is imperative to conduct further experiments employing knockout animal models to elucidate fully.

**Table 1:** Binding affinities and selectivities of oxytocin, WAY 267,464, and TC OT 39 to oxytocin receptors (OTR), vasopressin receptor 1A (V_1a_R), and vasopressin V2 receptors (V2R)

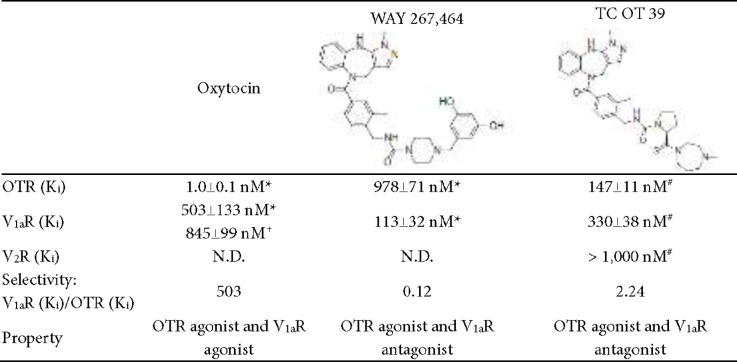

N.D.: Not detected

* Data were adopted from Hicks *et al*. (Hicks *et al*., 2012). Oxytocin and WAY 267,464 were evaluated for their Ki values for OTR and V1aR in isolated cell membrane preparations (stably expressed in HEK293 cells).

^#^ Data were adopted from Frantz *et al*. (Frantz *et al*., 2010). TC OT 39 was evaluated for its Ki values for OTR, V_1a_R, and V2R in isolated cell membrane preparations (stably expressed in CHO cells).

^+^ Data were adopted from Jurek and Neumann (Jurek and Neumann, 2018)

**Table 2:** Description of experimental groups of Sprague-Dawley (SD) rats subjected to the plantar test

Group	Sex	Intervention
M-Saline	Male	Male rats received an i.t. injection of 0.9% normal saline
F-Saline	Female	Female rats received an i.t. injection of 0.9% normal saline
M-WAY-267464 0.125 nanomole	Male	Male rats received an i.t. injection of WAY-267464 at a dose of 0.125 nanomole
M-WAY-267464 1.25 nanomole	Male	Male rats received an i.t. injection of WAY-267464 at a dose of 1.25 nanomole
M-WAY-267464 12.5 nanomole	Male	Male rats received an i.t. injection of WAY-267464 at 12.5 nanomole
F-WAY-267464 12.5 nanomole	Female	Female rats received an i.t. injection of WAY-267464 at 12.5 nanomole
F-WAY-267464 50 nanomole	Female	Female rats received an i.t. injection of WAY-267464 at a dose of 50 nanomole
M-TC OT 39 12.5 nanomole	Male	Male rats received an i.t. injection of TC OT 39 at 12.5 nanomole
F-TC OT 39 12.5 nanomole	Female	Female rats received an i.t. injection of TC OT 39 at 12.5 nanomole
M- Atosiban 12.5 nanomole	Male	Male rats received an i.t. injection of atosiban at 12.5 nanomole
F- Atosiban 12.5 nanomole	Female	Female rats received an i.t. injection of atosiban at 12.5 nanomole
M- WAY-267464 12.5 nanomole+Atosiban 12.5 nanomole	Male	Male rats received an i.t. injection of WAY-267464 and atosiban at 12.5 nanomole
M- TC OT 39 12.5 nanomole+Atosiban 12.5 nanomole	Male	Male rats received an i.t. injection of TC OT 39 and atosiban at 12.5 nanomole
F- WAY-267464 12.5 nanomole+Atosiban 12.5 nanomole	Female	Female rats received an i.t. injection of WAY-267464 and atosiban at 12.5 nanomole
F- TC OT 39 12.5 nanomole+Atosiban 12.5 nanomole	Female	Female rats received an i.t. injection of TC OT 39 and atosiban at 12.5 nanomole

(A) Intrathecal injection of WAY 267,464 dose-dependently suppressed intraplantar carrageenan-induced hyperalgesia in adult male rats (*P<0.05; ^##, ++ ^*P<*0.01; ^###, +++^ *P<*0.001; when compared with that in the saline group). (B) Quantitative analyses of the area under the curve (AUC) of the results are shown in Figure 1A. AUC represents the overall severity of hyperalgesia (^***^ *P<*0.001; compared with that in the saline group)

(A) For comparison, the intrathecal injection of WAY 267,464 suppressed intraplantar carrageenan-induced hyperalgesia in adult male and female rats. (B) The quantitative analyses of the area under the curve (AUC) of the results are shown in Figure 2. The AUC represents the overall severity of hyperalgesia (*** *P<*0.001; when compared with that in the corresponding saline group; ^###^ *P<*0.001; when female compared with that in the male group)

(A) Intrathecal injection of TC OT 39 suppressed intraplantar carrageenan-induced hyperalgesia in adult male and female rats for comparison. (B) The quantitative analyses of the area under the curve (AUC) of the results are shown in Figure 3. The AUC could represent the overall severity of hyperalgesia (*** *P<*0.001; when compared with that in the corresponding saline group)

(A)(C) Intrathecal injection of WAY 267,464 or TC OT 39 suppressed intraplantar carrageenan-induced hyperalgesia in adult male rats for comparison. (B)(D) The quantitative analyses of the area under the curve (AUC) of the results are shown in Figures 4A and 4C. The AUC represents the overall severity of hyperalgesia (*** *P<*0.001; when compared with that in the corresponding saline group; ^###^ *P<*0.001; when the co-administration of the atosiban group was compared with the WAY 267,464 or TC OT 39 group)

(A)(C) Intrathecal injection of WAY 267,464 or TC OT 39 suppressed intraplantar carrageenan-induced hyperalgesia in adult female rats for comparison. (B)(D) The quantitative analysis of the area under the curve (AUC) of the results shown in Figures 5A and 5C. AUC represents the overall severity of hyperalgesia (*** *P<*0.001; when compared with that in the corresponding saline group; ^###^ *P<*0.001; when the co-administration of the atosiban group is compared with the WAY 267,464 or TC OT 39 group)

## Conclusion

In summary, it was demonstrated that non-peptide OTR agonists, WAY 267,464 and TC OT 39, caused clear anti-hyperalgesia intrathecally in both sexes of rats. In addition to the anxiolytic effects (30), WAY 267,464 and TC OT 39 are good candidates for novel compounds in developing new drugs for treating inflammatory hyperalgesia. A previous study also found that a single intraperitoneal (IP) administration of LIT-001, a first non-peptide full agonist of OTR, also caused clear anti-hyperalgesia in male rats (31). Hence, these two compounds were shown to have great potential in future clinical applications. However, in contrast to intraperitoneal administration, intrathecal administration has certain clinical limitations and is less commonly used. Nonetheless, it still holds potential for application. Being effective in both sexes of animals, we predict the future nonrestrictive use of these compounds in clinics. Although the effective dose is higher than that of oxytocin, it can provide a stable and strong anti-hyperalgesia with no sex difference. Moreover, non-peptide agonists also have a higher half-life due to the prevention of degradation by peptidases.

Because WAY 267,464 and TC OT 39 both exhibited anti-analgesia effects in rats of both sexes, the hypothesis of the role of IRAP in sex difference was further supported. In addition, we have reported that oxytocin (i.t.) attenuates neuropathic allodynia in mice that underwent partial sciatic nerve ligation (7). We found that oxytocin’s effect in reducing neuropathic allodynia varied by sex. Therefore, we anticipate that WAY 267,464 and TC OT 39 can induce clear anti-allodynia with no sex difference. This should be clarified in a future study, which will broaden the application of these compounds in pain management.

## Data Availability

The data that has been used is confidential. Data will be made available upon request.

## Declaration

The authors declare that no artificial intelligence (AI) tools or technologies were used in the conception, data analysis, writing, or editing of this manuscript.
